# Therapeutic role of voltage-gated potassium channels in age-related neurodegenerative diseases

**DOI:** 10.3389/fncel.2024.1406709

**Published:** 2024-05-17

**Authors:** Janire Urrutia, Ane Arrizabalaga-Iriondo, Ana Sanchez-del-Rey, Agustín Martinez-Ibargüen, Mónica Gallego, Oscar Casis, Miren Revuelta

**Affiliations:** ^1^Department of Physiology, Faculty of Medicine and Nursery, University of the Basque Country (UPV/EHU), Bilbao, Spain; ^2^Department of Otorhinolaryngology, Faculty of Medicine, University of the Basque Country, Bilbao, Spain; ^3^Department of Physiology, Faculty of Pharmacy, University of the Basque Country (UPV/EHU), Vitoria-Gasteiz, Spain

**Keywords:** ion channel, Alzheimer, Parkinson, spinocerebellar ataxia, Huntington, channelopathies

## Abstract

Voltage-gated ion channels are essential for membrane potential maintenance, homeostasis, electrical signal production and controlling the Ca^2+^ flow through the membrane. Among all ion channels, the key regulators of neuronal excitability are the voltage-gated potassium channels (K_V_), the largest family of K^+^ channels. Due to the ROS high levels in the aging brain, K^+^ channels might be affected by oxidative agents and be key in aging and neurodegeneration processes. This review provides new insight about channelopathies in the most studied neurodegenerative disorders, such as Alzheimer Disease, Parkinson’s Disease, Huntington Disease or Spinocerebellar Ataxia. The main affected K_V_ channels in these neurodegenerative diseases are the K_V_1, K_V_2.1, K_V_3, K_V_4 and K_V_7. Moreover, in order to prevent or repair the development of these neurodegenerative diseases, previous K_V_ channel modulators have been proposed as therapeutic targets.

## 1 Introduction

Ion channels are essential for life as they play a fundamental role in neuronal signaling, muscle contraction or even nutrient transport (Weaver and Wearne, 2008). Moreover, voltage-gated ion channels are responsible for membrane potential maintenance, homeostasis, electrical signal production and controlling the Ca^2+^ flow through the membrane (Moiseenkova-Bell et al., 2021).

Ion channels are macromolecular pores that control ion flux through the cell membrane and consequently the intracellular ion balance (Eren-Koçak and Dalkara, 2021). The pore opens with mechanical, chemical or electrical stimulus and consequently ion channels allow ions to flow into or out the cell. Voltage-gated ion channels respond to a change in cell membrane potential and are highly selective for a specific ion (Na^+^, K^+^, Ca2^+^ or Cl^−^) (Trimmer and Rhodes, 2004). Meanwhile, ligand-gated ion channels respond to specific neurotransmitters among other molecules and mechanical-gated ion channels to changes in the mechanical force on the membrane.

Within the group of ion channels, there is a superfamily of K^+^ ion channels. This family is divided into four main families; the calcium-activated (KCa) family, inward rectifier (Kir) family, two-pore domain family (K2P) and voltage-gated (K_V_) family (Ocaña et al., 2004; Luo et al., 2021).

The KCa family is formed by 3 members, classified by single-channel conductance. Thereby, KCa1.1 (known as BK channels) shows large conductance, KCa3.1 (IK) intermediate and KCa2.1–3 (SK) small conductance (Sforna et al., 2018). KCa channels are expressed in neurons and other cell types in the central nervous system (CNS). Furthermore, the K_ir_ channels family is formed by 7 subtypes of channels (K_ir_1–K_ir_7), and each one has different members. They have an inward-rectification property that permits K^+^ enter the cell regulating membrane potential. These channels are expressed in different cells and regions of the CNS, and they regulate the hyperpolarization of the membrane potential and excitability (Akyuz et al., 2022). Meanwhile, two-pore domain K^+^ channels family consists of 15 members (K2P1–K2P7, K2P9–K2P10, K2P12–K2P18). K2P channels are dimers and in the CNS regulates cell excitability and maintains cellular resting potential. Some of the members are implicated in pathological conditions such as stroke, epilepsy, depression or inflammation (Talley et al., 2003).

### 1.1 K_V_ channels

But among all ion channels, the key regulators of neuronal excitability are the voltage-gated potassium channels (K_V_), the largest family of K^+^ channels (Shah and Aizenman, 2014). These K_V_ channels are divided into 12 subfamilies, named as K_V_1–K_V_12. They are composed of 4 α-subunits, each one containing 6 α-helical transmembrane domains (S1–S6), voltage sensor (S1–S4) and the ion pore (S5–S6). The N- and C-terminals are intracellular and they have different regulation sites. They differ in biophysical and pharmacological properties and in auxiliary β-subunits too, that modulate their activity, trafficking and location (Kuang et al., 2015). Although some channels regulate neuronal excitability, others participate also in the duration of cardiac action potentials and are involved in cell proliferation or even cancer (Bachmann et al., 2020).

Voltage-gated potassium channels are transmembrane channels responsible for returning the depolarized cell to a resting state after an action potential (Gazulla and Berciano, 2023). Therefore, K_V_ channels are important modulating neuronal excitability in the CNS, but also participate regulating other organs function.

Changes in ion physical function or gaining or depletion of channel function results in channelopathies, several of them associated to neurodegenerative disorders (Orfali et al., 2024). In this review, we will describe the role of some voltage-gated potassium channel in age related neurodegenerative disorders and their modulation for these diseases therapy.

## 2 Age related neurodegenerative diseases and voltage-gated K^+^ channel modulation

Age related neurodegenerative diseases have common organ deterioration mechanisms due to ROS production and Ca^2+^ intracellular accumulation, inflammatory response and apoptosis that results in neuron loss and functional failure. There are some therapeutic options; however, these are limited. Several strategies have been explored during the last decades in order to palliate or reduce the symptoms (Trombetta-Lima et al., 2020). Potassium channels are able to modulate activity patterns, defining their vulnerability to degenerate and their physiological functions (Duda et al., 2016). K_V_ channels regulate cell excitability and homeostasis so they can be considered as therapeutic targets in order to prevent or reduce age related neurodegenerative diseases, since it has been reported that aging itself can affect these channels function. Because ROS levels are highly elevated in the aging brain, K^+^ channels might be affected by oxidative agents and be key in aging and neurodegeneration processes (reviewed in Sesti et al., 2010). In this condition, molecules involved in the redox balance could modify the channel function (Sahoo et al., 2014).

### 2.1 K_V_ channels in Alzheimer disease

**Figure 1:**
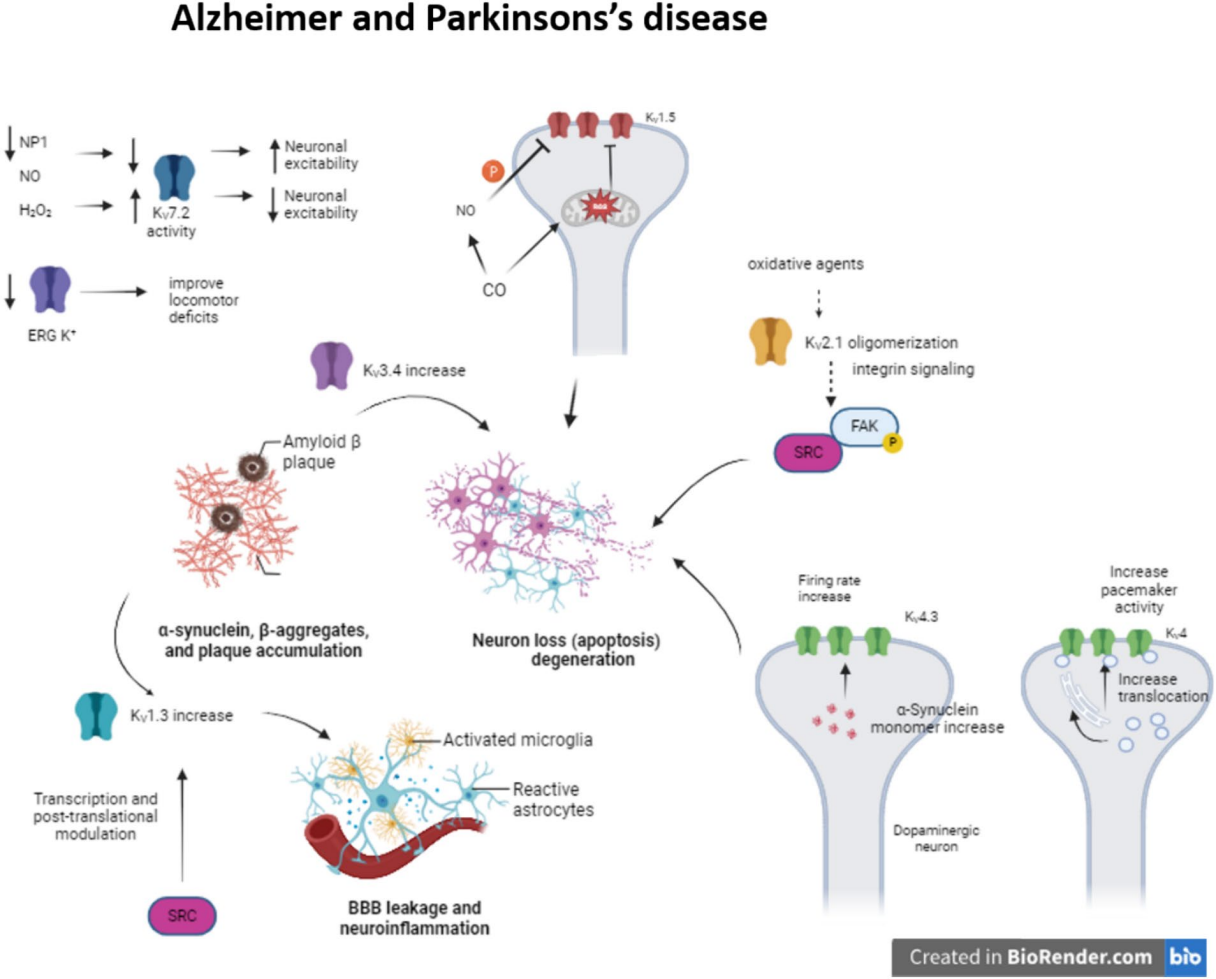
Modulation of K_V_ channels in Alzheimer and Parkinson’s disease. The alteration of some K_V_ channels expression or function modifies neuron excitability arising neuron apoptosis and neuroinflammation in Alzheimer and Parkinson’s disease.

During Alzheimer disease (AD), the amyloid β-peptide (Aβ) deposition causes synaptic dysfunction and consequently neuronal loss. There have been identified several K_V_ channels that can regulate the firing rate (K_V_1, K_V_4 or K_V_7) or the duration of the action potential (K_V_2 or K_V_3) (Li, 2022) (Figure 1). Moreover, the impairment in these K_V_ channels is associated with several pathogenic mechanisms. For instance, K_V_3.4 expression increase due to Aβ deposition initiates apoptotic processes and K_V_2.1 contributes to potassium mobilization during neuronal apoptosis, so overexpression of this channel promotes this process (Sun et al., 2022). The formation of K_V_2.1 oligomers by oxidative agents contribute to neurotoxicity and this phenomenon is aggravated in AD models (Cotella et al., 2012; Wei et al., 2018). This oligomerization triggers integrin signaling, activating Src tyrosine kinases via autophosphorylated FAK (Yu et al., 2019).

Meanwhile, enhanced K_V_1.3 expression in microglia, a key regulator of microglia function and related to inflammatory response, after amyloid plaque formation produces proinflammatory cytokine release and apoptotic cascade (reviewed in Revuelta et al., 2022). K_V_1.5 is mostly studied in the heart, but it is also presented in the brain and its activity is associated with apoptosis. It has been seen that H_2_O_2_ increases channel activity (Caouette et al., 2003). In contrast, CO inhibits K_V_1.5 current by the increase of ROS, which directly regulates the channel. Besides, the increase of NO in response to CO inhibits the channel activity by channel phosphorylation (Al-Owais et al., 2017).

Concerning K_V_4 channelopathies they have been linked to AD, schizophrenia and epilepsy (Cercós et al., 2021). Particularly in AD, the expression of KChIP3 (K_V_ channel-interacting protein 3 or calsenilin) is increased. This KChIP3 mechanically promote the translocation of K_V_4 channels to cell membrane, modulating the pacemaker activity (Buxbaum, 2004; Wu et al., 2023). They are associated with presenilins (PS1 and PS2), transmembrane proteins that are related to early-onset familial AD (Bähring, 2018) KChIP3 also modifies the gating of the channel, delaying the kinetic inactivation and accelerates the kinetic recovery from inactivation. Indeed, K_V_4.3 is involved in transient outward A type potassium current in neurons (Lopez-Hurtado et al., 2019).

The proapoptotic protein pentraxin (NP1) is another protein presented in dystrophic neurites in AD and related to the regulation of synapse density (Ma et al., 2018). This NP1 regulates the surface expression of K_V_7.2, a channel that controls neuronal excitability. K_V_7 channels generate M-current, slow voltage dependent outward current that contributes to the maintenance of the resting membrane potential, but can also exert a dampening effect on neuronal excitability. K_V_7.2 overexpression prevents cells from increased neuronal excitability and synapse, a situation provoked by NP1 downregulation during AD (Figueiro-Silva et al., 2015) (Table 1).

**Table 1:** Effect of different channelopathies in the neurodegenerative disease and channel modulator.

Associated pathology	K_V_ channel	Localization SNC	Channel expression during the disease	Function	Channel modulators	References
AD	K_V_1 (K_V_1.3)	Brain (oligodendrocytes, microglia)	Upregulated	Neuroinflammation	PAP-1, BmKTX	Wang et al. (2020)
	K_V_2 (K_V_2.1)	Brain (cortex and hippocampus)	Upregulated	Neuronal apoptosis	Tacrine	Wei et al. (2018)
	K_V_3 (K_V_3.4)	Brain (brainstem, hippocampal granule cells)	Upregulated	Neuronal apoptosis	BDS-I	Sun et al. (2022)
	K_V_4	Brain, cochlear nucleus	Upregulated	Neuroexcitation	Repaglidine, CL-888	Bähring (2018)
	K_V_7 (K_V_7.2)	Brain, neuroblastoma	Downregulated	Neuroexcitation	Retigabine	Figueiro-Silva et al. (2015)
PD	K_V_1 (K_V_1.3)	Brain (oligodendrocytes, microglia)	Upregulated	Neuroinflammation	PAP-1, BmKTX	Sarkar et al. (2020)
	K_V_4 (K_V_4.3)	Brain (hippocampal and cortical pyramidal neurons)	Upregulated	Neuroexcitation	Repaglidine, CL-888	Chen et al. (2018)
	K_V_7	Brain, brainstem auditory nuclei, neuroblastoma	Upregulated	GABAergic and DA neurons firing properties modulation	Retigabine, XE991	Hansen et al. (2006)
HD	K_V_2 (K_V_2.1)	Brain (cortex and hippocampus)	Downregulated	Synaptic dysruption	Tacrine	Zhang et al. (2018)
	K_V_4.3	Brain (hippocampal and cortical pyramidal neurons)	Downregulated	Neuroprotection	Repaglidine, CL-888	Lopez-Hurtado et al. (2019)
SCA	K_V_3.3 (*KCNC3*)	Brain, purkinje cells, motoneurons; auditory brainstem; cerebellar neurons	Gene mutation	SCA13	Genetic inactivation with antisense oligonucleotides (ASOs)	Zhang and Kaczmarek (2016)
	K_V_4.3 (*KCND3*)	Brain (hippocampal and cortical pyramidal neurons)	Gene mutation	SCA19	Repaglidine, CL-888	Zanni et al. (2021)

### 2.2 K_V_ channels in Parkinson disease

The neuropathological characteristics of Parkinson’s disease (PD) (Figure 1) are the degeneration of dopaminergic neurons in the CNS and the presence of Lewy bodies, α-synuclein-(SNCA)-positive intracytoplasmic inclusions (Poewe et al., 2017). Moreover, in PD pathophysiology there is inflammation due to microgliosis and astrogliosis and it seems that this inflammation is crucial for PD progression (Table 1) (Tansey and Goldberg, 2010).

It has been described that K_V_1.3 expression is upregulated in some animal models of PD, *in vitro* experiments, and postmortem human PD brains. Fyn, the Src family kinase that is involved in the microglia activation (Panicker et al., 2015), could regulate the K_V_1.3 channel expression both transcriptionally and post-translationally modifying its activity and therefore increasing neuroinflammation (Sarkar et al., 2020).

A-type K^+^ current, generated by K_V_4.3 and KChip3 interaction, is present in CNS DAergic neurons that contribute to regulating the neuron’s tonic activity (Chen et al., 2018). A53T-SNCA mice mutant which overexpress human α-synuclein with a PD-associated mutation (A53T), showed a oxidative dysfunction of this current induced by the overexpression of the α-synuclein, increasing the firing rate frequency of the dopaminergic substantia nigra neurons (Subramaniam et al., 2014); in both PD animal models and PD patients K_V_4.3 expression changes have been observed.

K_V_7 channels are expressed in GABAergic and Dopaminergic neurons in the striatum. Activation of K_V_7 channels induces hyperpolarization of Dopaminergic neurons and inhibits the excitatory activity (Hansen et al., 2006). Four out of five (K_V_7.2–K_V_7.5) M-channels members’ activity is regulated by oxidative and nitrosylation processes in sensory neurons. While oxidation by H_2_O_2_ augmented channel activity (Linley et al., 2012), nitrosylation by NO donors inhibited it (Ooi et al., 2013). In oxidative-stress-induced neurodegeneration model, oxidation of the S2–S3 linker of the K_V_7 enhance the M-current, protecting cells due to neuronal silencing (Gamper et al., 2006; Nuñez et al., 2023).

During PD there is a progressive loss of dopamine (DA) in substantia nigra and consequently in the striatum. Recent studies have proposed the therapeutic role of KCNQ channel blockers as they increase the neuronal bursting pattern in the substantia nigra and enhance DA synthesis in the striatum (Liu et al., 2018).

ERG or Kv11 K^+^ channels are present in the locus coeruleus (LC) of the brain. This area is related to cognition, learning and memory, among other roles (Uematsu et al., 2017; James et al., 2021; Dahl et al., 2022). This channel prevents increased firing rate and discharge irregularities in those LC neurons (Hasan et al., 2022). In PD, the LC neurons degeneration is present before DAergic neurons degeneration. It has been seen that in Parkinsonian rats the use of ERG K^+^ channels blockers improves the locomotor deficits, whereas the activators do the opposite, increase burst mode and impaired motor function (Huang et al., 2017). So, this channel dysfunction could be implicated in PD.

### 2.3 K_V_ channels in Huntington disease

**Figure 2:**
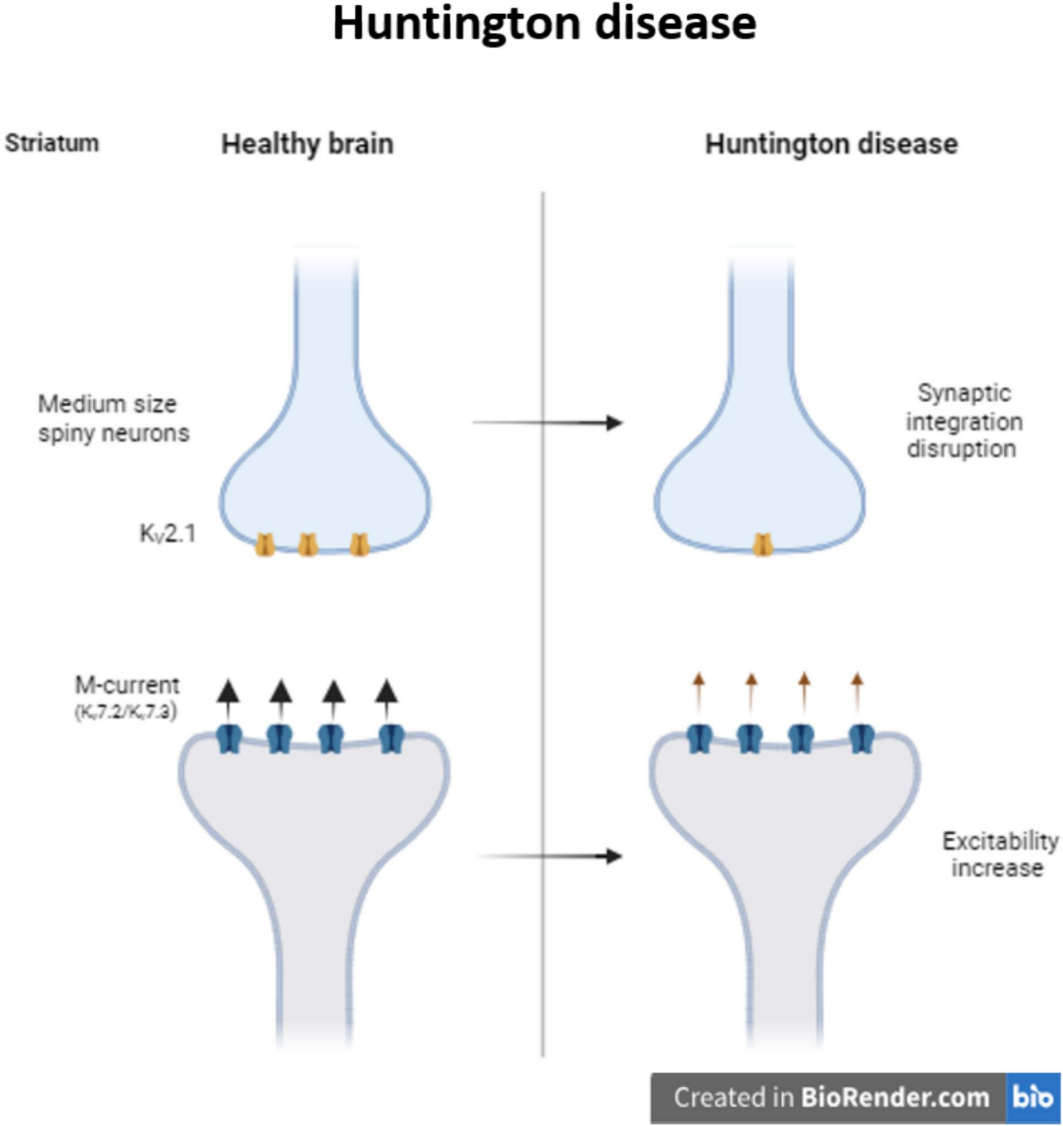
Modulation of K_V_ channels in Huntington disease. Decrease in the expression of K_V_2.1 provokes synaptic integration disruption in medium size spiny striatum neurons. In Huntington patients, the decrease in the M-current increase the excitability of the striatum neurons.

Huntington disease (HD) is a progressive neurodegenerative disease caused by the CAG triples expansion in the Huntington gene (MacDonald et al., 1993). Neurons from striatum and the cerebral cortex are the two main regions affected during HD. Particularly in the medium size spiny neurons (MSNs) from the striatum, K^+^ channels are necessary to maintain the membrane potential hyperpolarized and the slow firing rate. During HD there is a reduction of K_V_2.1 channel in MSNs disrupting synaptic integration and consequently information processing (Zhang et al., 2018). At the same time, it is also reported a reduction of M-current, reducing the control of the excitability in striatal output neurons of R6/2 mice (Figure 2). Retigabine, a potential antiepileptic drug, not only restores the hyperactivity network, but also improves motor skills of these mice (Cao et al., 2015).

Moreover, the previously mentioned KChIP3 is downregulated in HD patients and is associated with neuroprotection (An et al., 2000). Several K_V_4.3/KChIP3 channel complex modulators have been proposed in the last years as therapeutic targets to modulate channel gating and promote neuroprotection during HD (Lopez-Hurtado et al., 2019).

### 2.4 K_V_ channels in spinocerebellar ataxia

Spinocerebellar ataxia (SCA) is an autosomal dominant neurodegenerative disorder characterized by progressive ataxia with variable symptoms. There are more than 40 distinct genetic SCA (Bhandari et al., 2024). In humans, K_V_ channelopathies are linked to disorders in cell excitability, but only few are the principal responsible for neurodegeneration, mostly the ones that produce spinocerebellar ataxias (SCA) (Zhang and Kaczmarek, 2016). In 2002 there was identified a mutation in the KCND3 gene, that codifies K_V_4.3 channel that causes SCA19 (Verbeek et al., 2002). Mutations in this gene provoke impairments in the channel traffic from the endoplasmic reticulum to Golgi membrane, reducing the functionality of the channel and consequently provoking the disorder (Duarri et al., 2012; Zanni et al., 2021).

In the mouse model of SCA3, altered K_V_ channel function is associated with Purkinje neuron dysfunction; specifically, the inactivation of K_V_3 current seems to be the cause (Shakkottai et al., 2011).

The SCA13 is another disorder produced by a mutation in the gene that encodes K_V_3.3 channel resulting in cerebellar neurodegeneration. The major function of this channel is to drive the repolarization phase of action potential, so mutations in this gene produce disorders of excitability and consequently cerebellar neurodegeneration (Rudy and McBain, 2001; Zhang and Kaczmarek, 2016).

Another K_V_ channel that has been linked to episodic ataxia type 1 (EA1) is the *KCNA1* (K_V_1.1) (Tan et al., 2013). A mutation in *KCNA1* gene is the only responsible for the EA1 resulting in episodic ataxia and myokymia. Mutations in this gene can modify the channel current density and consequently channel gating, provoking dysfunctions in the circuits located in several tissues, such as cerebellum, hippocampus or cortex in EA (D’Adamo et al., 2020).

## 3 Therapeutic approach

Since K_V_ channels are the main regulators of neuronal excitability, their up- and downregulation is linked to enhance several neurodegenerative disorders, such as AD, PD or even provoke ataxias. In order to prevent or repair the development of these neurodegenerative diseases, K_V_ channel modulators have been proposed as therapeutic targets (Table 1).

### 3.1 K_V_1 channel modulators

Although certain Na_V_-blocking anticonvulsant drugs (carbamazepine, phenytoin, and lamotrigine) are used to reduce seizures, they do not work as therapy. In this sense, K_V_1 (K_V_1.1 and K_V_1.3) channelopathies are involved in cell excitability and firing rates in diverse pathological processes, small molecule research and *in silico* approaches are currently being studied to find modulators of these channels as a target (D’Adamo et al., 2020). K_V_1.1 dysfunction, for instance, is responsible for episodic ataxia type 1 (EA1). In that regard, some negative modulators of K_V_1.1 have been found (Wacker et al., 2012), but no molecule able to specifically modulate K_V_1.1 channels has yet been described. Experimental studies have demonstrated that some resin acids generated by some plants (piramic acid and dehydroabietic acid) are able to open K_V_ channels *in vitro* by changing voltage-dependent activation towards negative potentials (Ottosson et al., 2015).

Further, hyperactivation of the mTOR pathway is involved in the increased expression and altered distribution of K_V_1.1 channels in the hippocampus of mice with cortical dysplasia with epilepsy. In those mice, the classical mTOR inhibitor rapamycin normalized the levels of K_V_1.1, thus proposing that the mTOR pathway may be another possible research target to modulate K_V_1.1 expression (Nguyen and Anderson, 2018).

On the other hand, the K_V_1.3 channel is considered a novel therapeutic target to treat neuroinflammatory disorders, such as PD and AD, as it plays a crucial role in microglial cells subsets (Wang et al., 2020). During these neurological disorders, there is an overexpression of K_V_1.3 channels concluding that K_V_1.3 specific blockers could mitigate neuroinflammation, and become specific therapeutic candidates during AD or PD (reviewed in Revuelta et al., 2022).

Some studies showed that PAP-1, a K_V_1.3 blocker, could reduce cerebral Aβ load, diminish neuroinflammation, enhance plasticity of hippocampal neurons and improve behavioral deficits in APP/PS1 transgenic mice (Maezawa et al., 2018). Furthermore, PAP-1 administration reduced neurodegeneration and neuroinflammation in animal models of PD (Sarkar et al., 2020). Moreover, it has been shown that some toxins produced by certain animals can act as a modulator of K_V_1.3 channels. Specifically, the effects of BmKTX, a scorpion toxin, targeting K_V_1.3 have been studied as a possible treatment of AD and PD, as it could block microglial activation and thus reduce the neuroinflammation (Wang et al., 2020).

### 3.2 K_V_2.1 channel modulators

K_V_2.1 channel overexpression promotes neurotoxicity and neuronal apoptosis in AD models (Sun et al., 2022), whereas in HD there is a reduction in these channels in medium-sized spiny neurons (MSNs) contributing a synaptic disruption (Zhang et al., 2018).

It has been described that tacrine, a cholinesterase inhibitor, can act on K_V_ channels. It reduces the expression of K_V_2.1 channels and increases cell proliferation providing neuroprotection during AD (Hu et al., 2020). AD-related mutations can promote increased ROS production leading to K_V_2.1 channel function loss. Therefore, inhibition of this channel could offer a novel therapeutic approach for AD (Frazzini et al., 2016).

Indeed, several studies relate the activation of K_V_2.1 channel activators with a better prognosis during HD, since these channels are downregulated in the disease and are associated with the mitochondrial oxidative stress generated in HD (Zhang et al., 2018).

### 3.3 K_V_3 channel modulators

K_V_3.3 channel dysfunction result in the SCA13. A recent study shows that the genetic suppression of K_V_3.3 channels using antisense oligonucleotides (ASOs) can reverse the SCA13 outcomes (Zhang et al., 2021), meaning that targeting K_V_3.3 expression may provide a potential therapeutic approach for SCA13.

Concerning the K_V_3.4 channel, its expression is upregulated during AD due to Aβ deposition, initiating neuronal apoptosis. Recent results suggest that rapid activation/inactivation of these channels could be involved in Aβ-induced neurotoxicity. Therefore, reducing the expression and/or function of K_V_3.4 in brains with AD could protect Aβ-mediated synaptic alterations (Yeap et al., 2022). Among K_V_3.4 targets, the BDS-I (blood depressing substance-I), a marine toxin extracted from *Anemonia Sulcate*, inhibits the channel activity, provoking a reduction of neuronal apoptosis, reducing the expression of certain stress markers, such as active caspase 12; preventing Aβ1-42 induced reactive oxygen species (ROS) production and decreasing the release of pro-inflammatory cytokines (Piccialli et al., 2021).

### 3.4 K_V_4 channel modulators

In the hippocampus, K_V_4 channelopathies are related to epilepsy, schizophrenia, and AD. Therefore, pharmacological modulation of somato-dendritic subthreshold-activating K^+^ current could function as a therapeutic target for these pathologies (Cercós et al., 2021).

Besides, as previously mentioned, KChIP3 is downregulated in HD patients, promoting neuroprotection. Hence, K_V_4.3/KChIP3 channel complex inhibitors have been proposed as potential therapeutic targets to promote neuroprotection during HD (Lopez-Hurtado et al., 2019). To date, some molecules, such as repaglidine and CL-888, have been shown to bind and inhibit K_V_4.3 currents.

### 3.5 K_V_7 channel modulators

In addition to their well-known relation with infantile epileptic encephalopathies, K_V_7 channelopathies are also linked to several age related neurodegenerative diseases (including AD and PD), such as neurotoxicity and alteration of GABAergic and Dopaminergic neuron activation properties. Downregulation of K_V_7.2 provokes neurotoxicity in AD and therefore, finding activators of these channels could be a therapeutic approach to increase neuronal excitation and synapse. Among other drugs, retigabine, has been described as capable of increasing potassium K_V_7.2–7.3 channel currents (Czuczwar et al., 2010). Retigabine acts as a positive allosteric modulator, stabilizing the open form of these channels after binding to a hydrophobic pocket near the channel gate (Gunthorpe et al., 2012). Nevertheless, this drug is not in use due to side effects. Even so, this suggests that pharmacological modulation of the M-current could exert beneficial effects on the cognitive deficits involved in the pathophysiology of neurological disorders (Alles and Smith, 2021).

On the other hand, upregulation of K_V_7 channels causes a modulation of GABAergic and Dopaminergic neuron activation properties. In this sense, XE991 blocks KCNQ channels promoting action potential in DAergic neurons and increasing their excitability. Furthermore, XE991 enhances suprathreshold synaptic responses and promotes depolarization of striatal GABAergic projection neurons (Chen et al., 2018).

### 3.6 ERG channel modulators

K_V_11 or ERG channel could be another therapeutic target for PD since the modulation of subthalamic discharge by ERG channel inhibitors attenuates motor dysfunction in PD rats (Huang et al., 2017). The partial block of ERG K^+^ channels by antipsychotic drugs has also been linked to better dopaminergic neuronal excitability (Shepard et al., 2006).

## 4 Conclusion

K_V_ channels are essentials for a variety of cell functions. Some of these functions are related with the neuron excitability and it has been probed that the impairment of these channels are implicated in some neurodegeneration diseases. Taking these channels as therapeutic targets and modulating the function of this channel family could be promising to prevent some of the symptoms of these neurodegenerative diseases.

## Author contributions

JU: Data curation, Funding acquisition, Resources, Validation, Visualization, Writing – original draft, Writing – review & editing. AA-I: Conceptualization, Methodology, Resources, Writing – original draft. AS-d-R: Conceptualization, Validation, Writing – review & editing. AM-I: Conceptualization, Data curation, Supervision, Writing – review & editing. MG: Supervision, Writing – review & editing. OC: Supervision, Writing – review & editing. MR: Data curation, Funding acquisition, Supervision, Writing – original draft, Writing – review & editing.
